# Automated glioma detection and segmentation using graphical models

**DOI:** 10.1371/journal.pone.0200745

**Published:** 2018-08-21

**Authors:** Zhe Zhao, Guan Yang, Yusong Lin, Haibo Pang, Meiyun Wang

**Affiliations:** 1 Collaborative Innovation Center for Internet Healthcare, Software and Applied Science and Technology Institute, Zhengzhou University, Zhengzhou 450002, Henan, China; 2 School of Computer Science, Zhongyuan University of Technology, Zhengzhou 450007, Henan, China; 3 Department of Radiology, Henan Provincial People’s Hospital, Zhengzhou 450001, Henan, China; Institute of Automation Chinese Academy of Sciences, CHINA

## Abstract

Glioma detection and segmentation is a challenging task for radiologists and clinicians. The research reported in this paper seeks to develop a better clinical decision support algorithm for clinicians diagnosis. This paper presents a probabilistic method for detection and segmentation between abnormal tissue regions and brain tumour (tumour core and edema) portions from Magnetic Resonance Imaging (MRI). A framework is constructed to learn structure of undirected graphical models that can represent the spatial relationships among variables and apply it to glioma segmentation. Compared with the pixel of image, the superpixel is more consistent with human visual cognition and contains less redundancy, thus, the superpixels are considered as the basic unit of structure learning and glioma segmentation scheme. *ℓ*_1_-regularization techniques are applied to learn the appropriate structure for modeling graphical models. Conditional Random Fields (CRF) are used to model the spatial interactions among image superpixel regions and their measurements. A number of features including statistics features, the combined features from the local binary pattern as well as gray level run length, curve features, and fractal features were extracted from each superpixel. The features are then passed by *ℓ*_1_-regularization to ensure a robust classification. The proposed method is compared with support vector machine and Fuzzy c-means to classify each superpixel into normal and abnormal tissue. The proposed system is tested for the presence of low grade as well as high grade glioma tumors on images collected from BRATS2013, BRATS2015 data set and Henan Provincial People’s Hospital (HNPPH) data set. The experiments performed provides similarity between segmented and truth image up to 91.5% by correlation method.

## 1 Introduction

Brain tumors are abnormal and uncontrolled growth of cells in the body. Primary brain tumors do not spread to other body sites, and can be malignant or benign. The majority of primary brain tumours originate from glial cells (termed glioma) and are classified by their histopathological appearances using the World Health Organization (WHO) [1] system into low-grade glioma (LGG) (grades I and II) and high-grade glioma (grade III anaplastic glioma and grade IV glioblastoma) [2]. Tumors are classified based on the four properties for intra-tumoral regions, namely “edema,” “non enhancing (solid) core,” “necrotic (or fluid-filled) core,” and “active core.” Human experts indicate each segmentation map into three classes, namely the “whole” tumor (including all four tumor classes), the tumor “core” (including all tumor classes except “edema”), and the “active” tumor (containing the “active core” only) [3]. A Magnetic Resonance Imaging (MRI) provides information about the human soft tissue anatomy and helps to diagnosis of brain tumor. The combination of different sequences of MRI techniques, such as T1-weighted (T1-w) MRI and T2-weighted (T2-w) MRI, are used for monitoring and evaluating the brain tumors. Images from conventional T1-w and T2-w MRI, being characterized by a high contrast and spatial resolution [4].

Many segmentation techniques are available in the existing literatures. The region growing method is most commonly used and it is the simplest technique of region based segmentation. This method is utilized to obtain bonded region of pixels which are similar to the original image [5]. A single click ensemble segmentation (SCES) approach based on an existing “Click & Grow” algorithm [6], this method makes multiple seed points are automatically generated by the manual seed. Fuzzy-c means is a clustering method based on object function minimization that segments the data set into two or more clusters, each pixel is allocated a membership function value to the available classes based on its attributes [7]. Several modifications and extensions on fuzzy c-means are reported in the literature [8, 9]. The hybrid method are fast, robust, accurate which combines the advantages of two or more methods for brain tumour segmentation. In [10], Haralick features, high-order derivative maps, and Tamura features were extracted from each volumes of interest. Then the support vector machine and random forest are used to construct classifier. In [11], content-based active contour model is implemented and high-dimensional features are reduced using genetic algorithm (GA). GA with support vector machine (SVM) and artificial neural network are implemented for brain tumour classification and compared, but the complexity increases due to hybridization.

A desirable image segmentation framework may be the one that is able to flexibly incorporate various types of information and constraints, and solve image segmentation by using the Graphical Models [12, 13]. Markov random fields (MRF) is an undirected graphical model, which is often used for image segmentation [14]. Graph cuts [15] algorithm is a popular segmentation based on MRF. This algorithm on graphs is used to exact or approximate energy minimization in image segmentation. Hamamci et al. [16] proposed a variation of the original graph-cut method using cellular automata for solving the shortest-path problem iteratively. Zhu et al. [17] also combined EM segmentation with MRF regularization, but added a post-processing pipeline including thresholding and morphological operations. Subbanna et al. [18] presented a new iterative, multi-stage graphical model, which was designed to leverage the strengths of both a local, voxel-based MRF and a contextual, regional (non-lattice based) MRF, in order to penalize implausible regional labels and label combinations, while also attaining accurate boundaries.

The Conditional Random Fields (CRF) is firstly proposed by Lafferty et al [19]. It is another type of undirected graphical model that has become increasingly popular. In researches for image segmentation, it is important to estimate the posteriori probability distribution over label random variables given the observed image. Since the segmentation problem can be described as a CRF model. Previous researches has shown that CRF models are effective in image segmentation. Kumar et al. [20] present a Discriminative Random Fields (DRFs) that classify image regions by integrating spatial interactions among labels and the observed images. Lee et al. [21] used conditional random fields for spatial regularization after previous voxelwise SVM classification from multiple modalities. Zhang et al. [22] develop a unified graphical model in which they combined CRF and Byesian network. The combination statement is powerful enough to capture more complex and heterogeneous relationships among image entities. The CRF discribe the spatial relationship among image regions and measurement. The multi-layer BN capture causal dependencies among various image entities. Bauer et al. [23] segment tumor and healthy tissues including sub-compartments based on SVM classification with integrated hierarchical CRF regularization. They also made use of prior knowledge about tissue adjacency probabilities.

Most methods based on undirected graphical models for image segmentation rely on the assumption that the graph structure is known. This assumption however does not necessarily exist in natural image data. So learning structure of graph from observed image is still an important issue. Structure learning has motivated a number of methods for learning sparse graphical models.

The proposed method is compared with support vector machine and fuzzy c-means to classify each superpixel into normal and abnormal tissue. The proposed system is tested for the presence of low grade as well as high grade glioma tumors on images collected from BRATS2013, BRATS2015 data set and Henan Provincial People’s Hospital (HNPPH) data set. The experiments performed provides similarity between segmented and truth image up to 91.5% by correlation method.

The remainder of this paper is organized as follows. In section 2, we introduce the method with detailed description. The experiments and analysis are given in section 3. Section 4 discuss some existing problems. Finally, we come to a conclusion in section 5.

## 2 Image segmentation based on graphical models

Due to both its importance and difficulty, the problem of structure learning for undirected graphical models has attracted considerable attention. Structure learning has motivated many approaches for learning sparse graphical models. These methods differ in the use of optimization algorithms and the provision of theoretical guarantees [24–26].

For reduction of computational consumption, we consider using the superpixels based method in here. Superpixel is an irregular block of pixels that are adjacent to each other and have similar visual characteristics such as color, brightness, texture, and so on. Most of these small areas retain the effective information for further segmentation of the image and preserve the boundary information of the objects in the image well. Therefore, we consruct the graphical models with superpixel regions as nodes.

**Fig 1:**
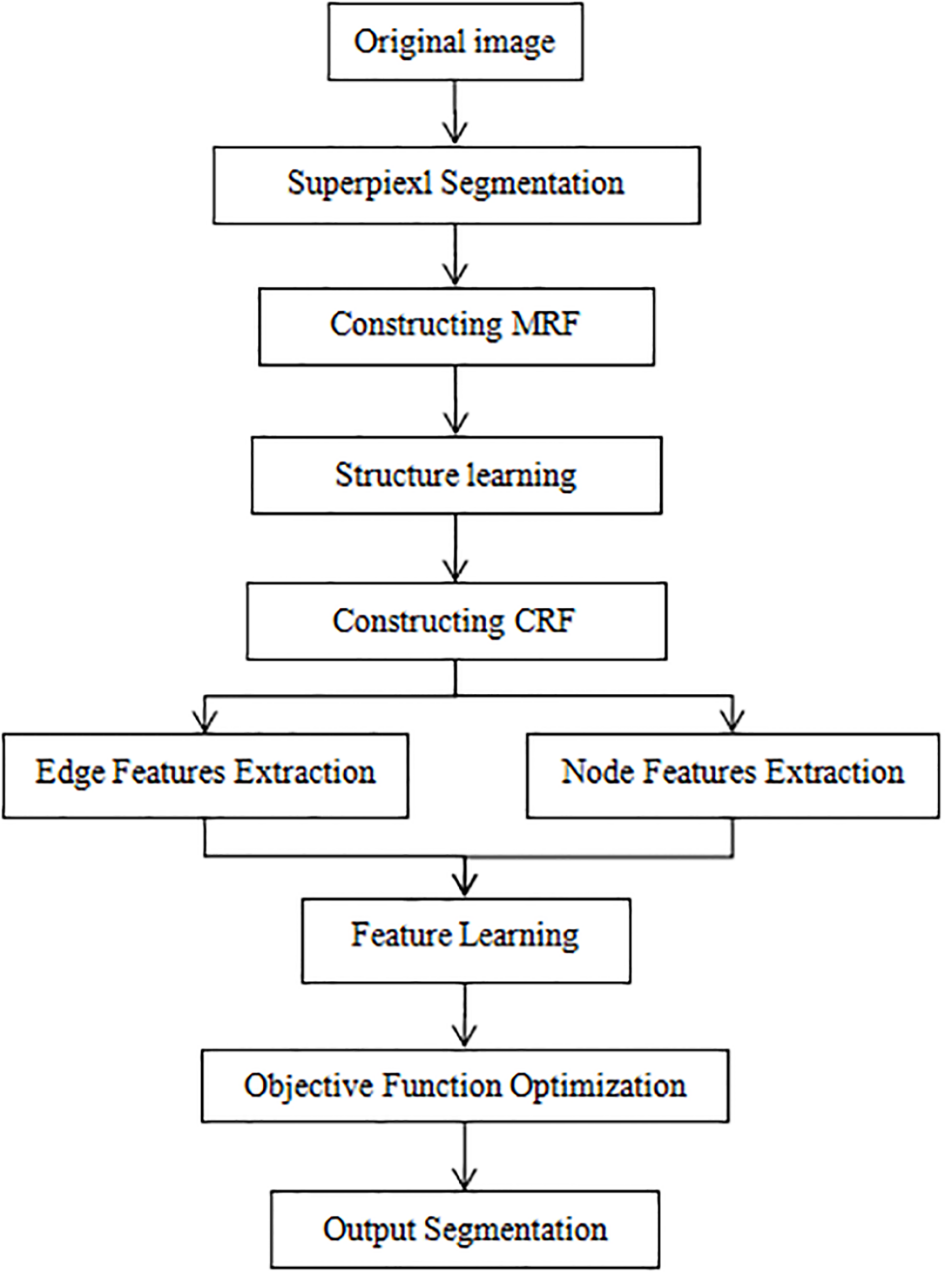
The flowchart of the proposed method.

In this paper, image segmentation can be thought of as a labeling problem under a probability maximization framework in graphical models. An important step therefore is to find the most likely configuration, or marginalization tasks that calculate the normalization constants or marginal probabilities. To do this, we propose a method for jointly learning the structure and parameters of undirected graphical models, formulating these tasks as a convex optimization problem. Instead of assuming a fixed underlying model, we select neighbours through a method based on regularized objective function, in which the neighborhood of any given node is estimated by performing function optimization subject to a *ℓ*_1_-constraint [27]. We consider *ℓ*_1_-regularization for each set of features associated with an edge, and formalize an efficient optimal method to find the globally optimal penalized maximum likelihood solution. After determining the structure of the graphical model, we construct CRF model through combining the superpixel region nodes and the image observation. The image segmentation based on CRF can be thought of as an optimization problem too. The flowchart of the proposed method is shown in Fig 1.

### 2.1 Structure learning

Many segmentations of images are required to estimate the parameters of the model of image labels, that is, the graph structure is known in advance. However, we are interested in both learning the graph structure and estimating the posterior distribution over labels. This is the key idea underlying the undirected graphical models which provide a powerful framework for statistical modeling. Undirected graphical models encode the conditional independency among the random variables through a sparse graph, and model the posterior distribution as a Gibbs field. Thus, both structure learning and posterior estimation play the important roles in image processing based on graphical models.

**Fig 2:**
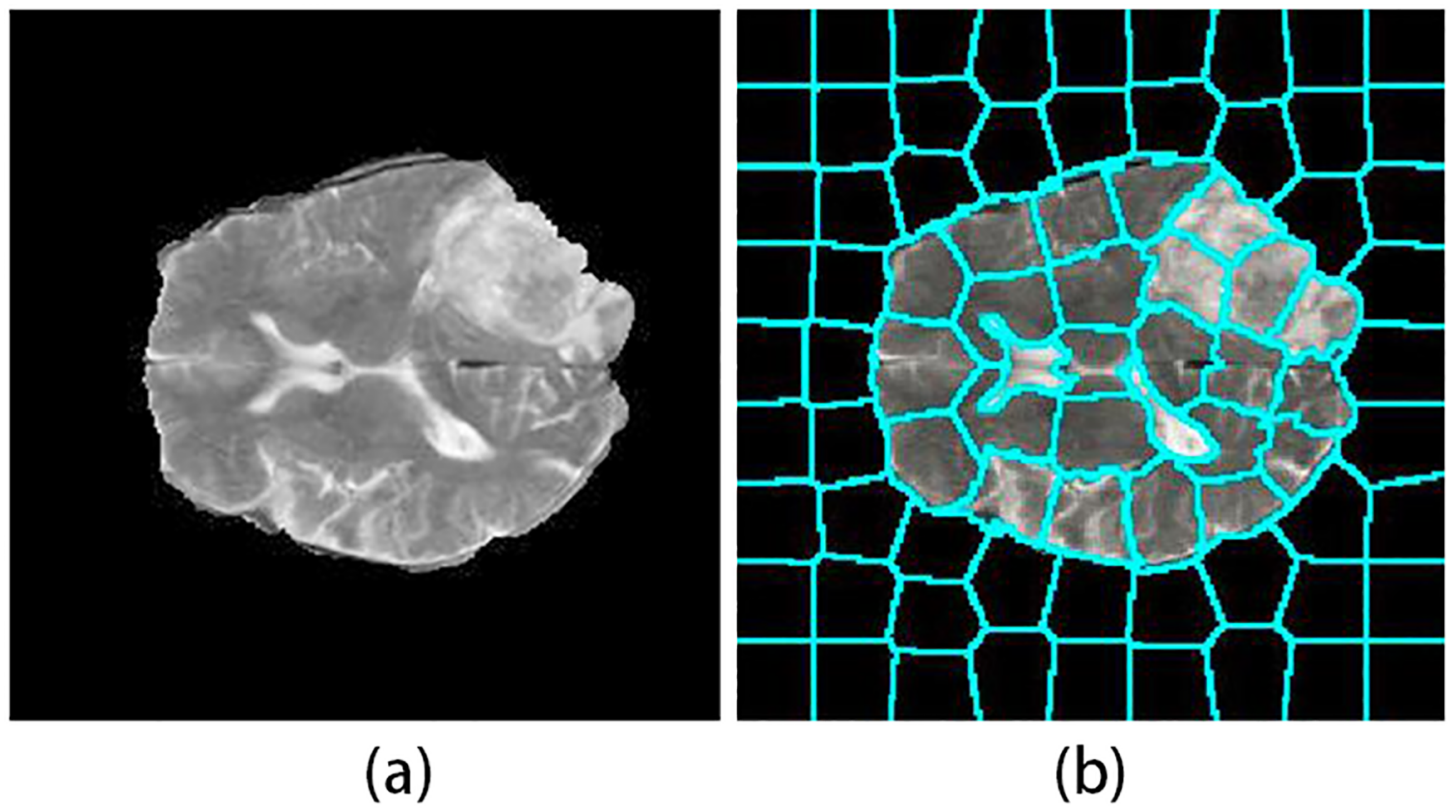
Superpixel image segmentation. (a) original image. (b) result of superpixel segmentation.

Superpixel image segmentation refers to the process of subdividing an image sub-region (also called superpixels). Compared with the pixel of image, the superpixel is more consistent with human visual cognition and contains less redundancy. Moreover, compact consistent superpixel segmentation can be used as a spatial feature for visual feature extraction. In addition, the superpixel undirected graphical models alleviate the computational burden that is a common problem in the models based on graphical model. Since the simple linear iterative clustering (SLIC) [28] is an popular method to generate an over-segmentation. The improved SLIC is used to achieve our goals, this improvement of SLIC algorithm to refine “Compactness” adaptively after the first iteration, thus the Superpixel block is more closely related to the tumor and edema region. Superpixel image segmentation is shown in Fig 2.

As shown in Fig 2, the image is first over-segmented into superpixel regions. Each superpixel region can be regarded as one node in the graphical models.

Structure learning is the problem of predicting a binary vector, each element of which indicates the presence or absence of a certain edge in a graph. Exploiting graph structure can significantly boost image processing performance. A Markov random field (MRF) is specified by an undirected graph. Its structure essentially reflects a priori knowledge, that is, which dependencies between variables need to be considered, while others can be ignored. Hence, it is also important to identify the structure of graph from image data. In the undirected graphical models based on the over-segmented image, we consider the superpixel as region node *i* that correspond to each superpixel (small image region) in the over-segmentation of original image. The absence of edge in the graph structure is a coding of the independence of random variables.

For convenience of computation, we only thought of the pairwise relationships among the region nodes. If there is a link between two superpixel regions, it means that there is an interaction between them, which is represented by the edge potential. In here, we consider the generalization of the Ising model [12] to represent probabilistic distribution of the pairwise MRF, the joint distribution take the following form

$$\begin{array}{c} P\left(y,\theta \right)=\frac{1}{Z(\theta )}\exp(\sum_{i\in V}\theta_{i}y_{i}+\sum_{i\in V}\sum_{j\neq i}\theta_{ij}y_{i}y_{j}), \end{array}$$ (1)

where in this case the *θ*_*i*_ is weight coefficient of node *i*, the zero weight *θ*_*ij*_ is equivalent to removing the edge from the node *i* and *j* and vice verse. *Z*(*θ*) is the normalizing constant, and its form is as follows,

$$\begin{array}{c} Z\left(\theta \right)=\sum_{{y_{i}}^{\prime }=1}^{p}\exp(\sum_{i\in V}\theta_{i}{y_{i}}^{\prime }+\sum_{i\in V}\sum_{j\neq i}\theta_{ij}{y_{i}}^{\prime }y_{j}), \end{array}$$

where *θ*_*i*_ is the parameter associated with state *s* for node *i*, and *θ*_*ij*_ is the edge parameter associated with state *s* for node *i* and *j*. Unlike the node potential, setting the specific variable to be zero in edge potential can influence the structure learning.

One important problem for such models is to estimate the underlying graph from *n* independent and identically distributed samples *y* = (*y*^(1)^, *y*^(2)^, …, *y*^(*n*)^) drawn from the distribution specified by some MRF. The structure learning of undirected graphical models usually use log-linear model which formalize a convex optimization problem in the parameter space. The optimization is performed effectively and is guaranteed to converge to global optimum. According to Eq (1), the symmetric negative pseudo-likelihood function for a set of *n* samples of *p*-vectors ***y***^*m*^ is given by

$$\begin{array}{c} \sum_{m=1}^{n}\sum_{i\in V}-\log p\left(y_{i}|y_{j\neq i},\theta \right). \end{array}$$ (2)

In order to estimate the parameter *θ* = {*θ*_*i*_, *θ*_*ij*_}, we consider the conditional distribution of *y_i_* given the other variables *y_j_*. A calculation shows that under the model Eq (1), *ϕ_i_*(*y_i_*, *θ_i_*) and *ϕ_ij_*(*y_i_*, *y_j_*, *θ_ij_*), this conditional distribution takes the following form

$$\begin{array}{c} p\left(y_{i}|y_{j\neq i},\theta \right)=\frac{\exp[2y_{i}\left(\theta_{i}+\sum_{j\neq i}\theta_{ij}y_{j}\right)])}{\exp[2y_{i}\left(\theta_{i}+\sum_{j\neq i}\theta_{ij}y_{j}\right)]+1}, \end{array}$$ (3)

the variable *y_i_* can be considered as the response variable in a logistic regression, in which all of the other variable *y_j_* are the covariates.

The structure of graph can be got through shrinking the weight coefficients of edge potentials. *ℓ*_1_-regularized likelihood encourage sparsity [27]. As a result, structure learning is to find the high probability fields in the sample space, to represent the fields with potential functions, and study its corresponding weights. Given an optimization algorithm with initial feature set, model selection is to choose an edge potential function with nonzero weights.

Similar to the [29], we consider the problem of structure learning with the objective function as the *ℓ*_1_-norm constrained convex optimization. which formed as a sum of two convex terms: one is assumed to be differentiable and convex, another is convex and possibly non-differentiable. Thus, we can thought of optimizing the following objective function to estimate parameters and select neighborhood.

$$\begin{array}{c} \sum_{m=1}^{n}\sum_{i\in V}-\log p\left(y_{i}|y_{j\neq i},\theta \right)+\lambda^{m}{∥\theta_{ij}∥}_{1}, \end{array}$$ (4)

where λ^*m*^ is a tuning parameter. The neighborhood estimate (parameterized by λ) is defined by the nonzero coefficient estimates of the *ℓ*_1_-regularized regression.

Lassplore method [30] applying Nesterov’s method [29] for smooth convex optimization, and using the adaptive line-search to tune the step size adaptively and meanwhile guarantees the optimal convergence rate. Compare to the other methods, this method has advantages for estimating log-liner models. Thus, it is used to solve the non-smooth optimization problem in here.

### 2.2 Image segmentation

Each superpixel region can be regarded as one node in the CRF model. Based on the image observation, superpixel regions and the graph structure learned earlier, the CRF model is then constructed. Image segmentation based on CRF can be considered to predict a label of a superpixel node. The solution is to model the conditional probabilistic distribution, that is, to construct optimization function. In this way, the problem of image segmentation is convert into the optimization of objective function.

#### 2.2.1 Conditional random fields construction

Let ***y*** = {*y*_1_, *y*_2_, …, *y*_*p*_} be the label random variables corresponding to all superpixels, where *p* is the total number of superpixels in an image. ***x*** = {*x*_1_, *x*_2_, …, *x*_*p*_} are the corresponding local feature vector extracted from the observed image. In image segmentation, each output variable *y_i_* is the label of the region node at position *i*, and each input variable *x_i_* contains various features about the region node at position *i*, such as color, brightness, texture and so on. After determining the structure of the graphical model, we must consider to predict a label vector ***y*** of random variables given an observed feature vector ***x***.

One natural way of solving this problem is to model the conditional probabilistic distribution *P*(***y***|***x***) directly, which is all that is needed for labelling. This is a CRF. CRF is a kind of undirected graphical model that defines a log-linear distribution over label vectors given a observation image. A CRF can be thought of undirected graphical model, or Markov random field. Globally conditioned on observation *x*, that is,

$$\begin{array}{c} p\left(y_{i}|x,y_{j},j\neq i\right)=P\left(y_{i}|x,y_{j},j\in N_{i}\right), \end{array}$$ (5)

where $N_{i}$ represents the neighborhood of *i*.

Compared with the MRF model, the CRF model weakens the conditional independence assumption of the observation. The image segmentation based on CRF can be considered as a labeling problem. We thus wish to generate a random variables ***y*** = {*y*_1_, *y*_2_, …, *y*_*p*_} which represents label configuration of superpixel given an observed image ***x***, that is, to assign a label to a superpixel. As can be seen from the result of structure learning in the previous section, the number of neighbors is different for different region nodes. This means that the interactions between nodes is stronger or weaker. According to the Hammersley-Clifford theorem, the CRF directly model the posterior distribution *P*(***y***|***x***) as a Gibbs field [31]. Thus, we assume that *P*(***y***|***x***) be the conditional probability of the set of superpixel label assignments ***y*** given the observed image:

$$\begin{array}{c} P\left(y|x\right)=\frac{1}{Z}\text{exp}\left\{\sum_{i\in V}\left[\psi_{i}\left(x,y_{i}\right)+\sum_{i\in V}\sum_{j\in N_{i}}\psi_{ij}\left(x,y_{i},y_{j}\right)\right]\right\}, \end{array}$$ (6)

where *ψ_i_*(***x***, *y_i_*) is node potential function, and *ψ_ij_*(***x***, *y_i_*, *y_j_*) is edge potential function. *Z* is partition function. Our CRF model is a superpixel based model. We can infer the label for each superpixel from image measurements by using the CRF model Eq (6).

Similar definitions of the unary potentials of *ϕ*_*i*_ in the previous section. The following form of superpixel node potential function can be given,

$$\begin{array}{c} \psi_{i}\left(x,y_{i},\theta_{i},\tau_{i}\right)=\sum_{s=1}^{k-1}\left\{\left[\theta_{i}+\tau_{i}\varphi_{i}\left(x\right)\right]y_{i}\right\}, \end{array}$$

where *θ*_*i*_ is a set of bias parameters, and *τ*_*i*_ is a set of parameters associated with state *s* for region node *i*. *φ_i_*(***x***) is a function that maps the observations ***x*** on a feature vector. *δ*(⋅) denotes an indicator function that returns a value of 1 if its argument is true and 0 otherwise. In a similar fashion, we can write edge potential function $\psi_{ij}\left(y_{i}^{\prime },y_{j}^{\prime },\tau_{ij}\right)$ as

$$\begin{array}{c} \psi_{ij}\left(x,y_{i},y_{j},\theta_{ij},\tau_{ij}\right)=\sum_{s=1}^{k}\left\{\left[\theta_{ij}+\tau_{ij}\varphi_{ij}\left(x\right)\right]y_{i}y_{j}\right\}, \end{array}$$

where *θ*_*ij*_ is a set of bias parameters, and *τ*_*ij*_ is a set of parameters associated with state *s* for edge between *i* and *j*. *φ_ij_*(***x***) is a edge feature vector that can be used to model the relations between pairs of sites.

#### 2.2.2 Features description

Our image features *φ_i_*(***x***) consists of four classes of features, namely statistical features, texture features, curvature features, and fractal features. The explanation of the features used for the image segmentation applications is given in this section.

First-order intensity statistics [32] are referred as pixel intensity-based features. They express the distribution of grey levels within the selected region of interest (ROIs) which are the superpixels in our work.

Since brain tissues have complex structures, the intensity features are not sufficient for accurate segmentation of tumour, we use texture features to improve the accuracy of tumor segmentation. Gupta et al. proposed a combined strategy called Run Length of Centralized Patterns (RLCP) [33]. In this method, local binary pattern (LBP) [34] code is indexed and gray level run length (GLRL) [35] matrix in principal directions are formed to count occurrences of runs length for each gray level.

Image curvature is a shape-based feature which is computed by the gradients along x and y directions of an image, namely *f*_*x*_, *f*_*y*_, *f*_*xx*_ and *f*_*yy*_ are the second derivatives of the image intensity *I*(*x*, *y*), the two-dimensional curvature of the image is calculated as [36]:

$$\begin{array}{ccc} Curv & = & \frac{f_{xx}f_{y}^{2}+f_{yy}f_{y}^{2}-2f_{xx}f_{x}f_{y}}{{\left(f_{x}^{2}+f_{y}^{2}\right)}^{\frac{3}{2}}}. \end{array}$$ (7)

**Fig 3:**
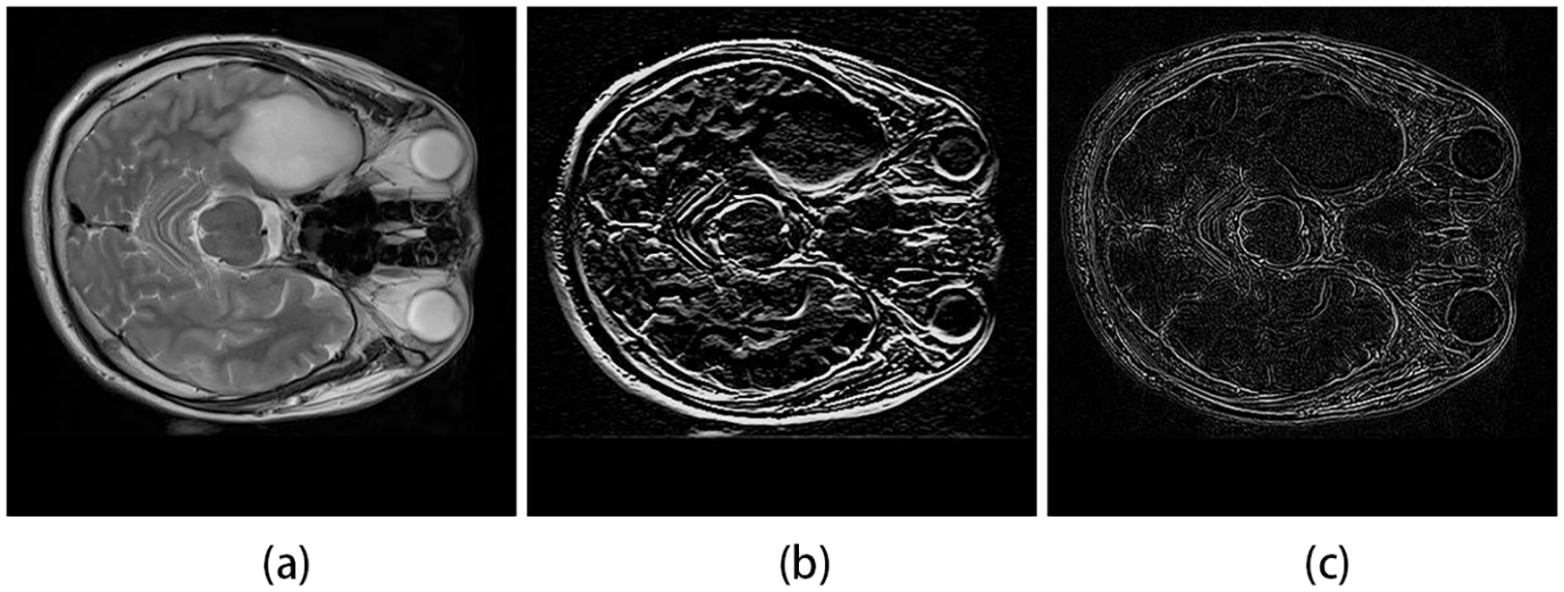
Gradients mapping and curvature mapping. (a)Original image. (b) Gradients mapping. (c) Curvature mapping.

The curvature feature for each superpixel is the average of the curvature values for all the pixels in the superpixel. Fig 3 shows the gradients mapping and curvature mapping for original image.

Due to the heterogeneity of glioma, tumor tissue has irregularity and complex structure in medical imaging, with the characteristics of fractal features [37] there is a good distinction between tumor area and normal tissue under certain scale. In this study, four thresholds binary images is obtained from quantitatively image by Otsu algorithm [38], and each binary image provides area, mean intensity and fractal dimension features. Fractal dimension features ia obtained by box-counting algorithm in [39].

#### 2.2.3 Parameter optimization

Let *ω* = {*ω*_*i*_, *ω*_*ij*_}, *ω*_*i*_ = {*θ*_*i*_, *τ*_*i*_}, *ω*_*ij*_ = {*θ*_*ij*_, *τ*_*ij*_}, *f_i_*(***x***) = {1, *φ_i_*(***x***)}, and *g_ij_*(***x***) = {1, *φ_ij_*(***x***)}. In order to estimate the parameter *ω*, we consider the conditional distribution of *y_i_* given the other variables *y_j_*. A calculation shows that under the model Eq (6), *ψ*_*i*_ and *ψ*_*ij*_, this conditional distribution takes the following form

$$\begin{array}{c} p\left(y_{i}|x,y_{j\in N_{i}},\omega \right)=\frac{\text{exp}\{2y_{i}\left[\omega_{i}^{T}f_{i}\left(x\right)+\sum_{j\in N_{i}}\omega_{ij}^{T}g_{ij}\left(x\right)y_{j}\right]\}}{\sum_{y_{i}^{\prime }}\text{exp}\left\{2y_{i}^{\prime }\left[\omega_{i}^{T}f_{i}\left(x\right)+\sum_{j\in N_{i}}\omega_{ij}^{T}g_{ij}\left(x\right)y_{j}\right]\right\}}, \end{array}$$ (8)

the variable *y_i_* can be considered as the response variable in a logistic regression in which all of the other variable *y_j_* are the covariates. Moreover, many features are extracted from image, however, not all of the features are what we want. The *ℓ*_1_-regularization of the node feature parameters can help us choose the appropriate features. Thus the image can be more accurately described. Finally, we can consider minimizing the following pseudo-likelihood with *ℓ*_1_-regularization of the node feature parameters, and *ℓ*_2_-regularization of the edge parameters to estimate parameters.

$$\begin{array}{c} \sum_{m=1}^{n}\sum_{i\in V}-\log p\left(y_{i}|x,y_{j\in N_{i}},\omega \right)+\lambda_{1}^{m}∥[\omega_{i}{∥}_{1}+\lambda_{2}^{m}∥[\omega_{ij}{∥}_{2}. \end{array}$$ (9)

The negative penalized logarithm of objective function in Eq (9) is convex with respect to the model parameters, and the minimum value can be obtained by gradient descent.

## 3 Experiments and analysis

In order to demonstrate the effectiveness of the proposed method, we evaluate the performance of the proposed method on a publicly available BRATS 2013 clinical data set and BRATS 2015 clinical dataset [3]. Thirty patients datasets and 50 synthetic datasets including ground truths are available in BRATS 2013, and BRATS 2015 contains 220 high grad(HGG) and 54 low grade (LGG) glioma MRI images including ground truths are provided by a trained human expert. We also further evaluate the performance of the proposed method on 161 patients sampled from Henan Provincial People’s Hospital (HNPPH), which is also provided ground truths by experienced radiologist. And all the patients provided their written informed consent for the use of their medical records. Only Henan Provincial Peoples’ Hospital authors Meiyun Wang had access to identifying information during preparation of this manuscript. For all dataset of each patient data, T1-w, T2-w, fluid-attenuated inversion recovery (FLAIR) and post-gadolinium T1-w MR images are available. Moreover, the research of this paper has been approved by the Life Science Ethical Review Committee of Zhengzhou University.

Our experiments can be designed in two groups. One group is conducted to test the task of structure learning of undirected graphical model, and the other is designed to evaluate the performance of image segmentation based structure learning. Both groups can be tested on various medical image data sets.

### 3.1 Structure learning

In our work, we first test the proposed model for learning structure of undirected graphical models using the BRATS data set for further segmenting images. The modification of SLIC can be used to get over-segmented image, in which each superpixel is thought of as a region node *i*. We will learn the structure of undirected graphical models, which can represent the spatial relationships among label variables *y_i_*, and assume that the variables *y_i_* follow the Markovian property. In general, the neighborhood system of the MRF is assumed to be known in advance. If two superpixels are adjacent, they are connected to each other, which is caused by the irregular shape of superpixel nodes. Different from the previous traditional case, the neighborhood structure in this paper is obtained by structure learning. We can consider minimizing the objective function Eq (4) to estimate parameters and select neighborhood.

**Fig 4:**
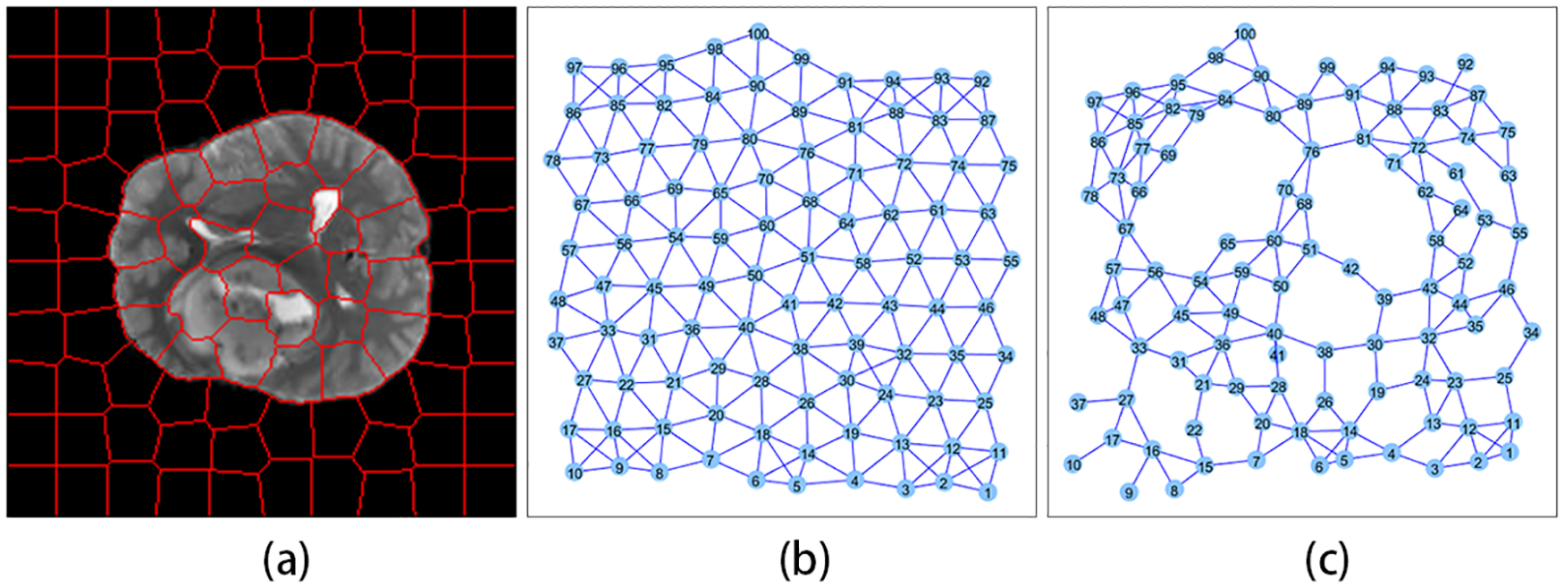
Results of structure learning. (a) Result of superpixel segmentation. (b) Graph structure based on adjacent superpixel nodes. (c) Structure learning result based on the proposed method where λ = 6.

Specifically, we formulate the objective function as the *ℓ*_1_-norm constrained convex optimization, which is continuously differentiable, and the problem domain set is closed and convex. The Nesterov’s method is used to solve this optimization problem because it has the optimal convergence rate among the first-order methods. The adaptive line search scheme allows to tune the step size adaptively and meanwhile guarantees the optimal convergence rate. Therefore, it is used to estimate an appropriate step size in each optimization of Nesterov’s method. The neighborhood structure obtained by structure learning is shown in Fig 4.

Fig 4(a) is a result of superpixel segmentation. In general, the adjacent region nodes can be regarded as neighbors in MRF based on superpixel. This neighborhood structure is shown in Fig 4(b). In this paper, the graph structure can be learned through minimizing logarithm of objective function Eq (4). One edge corresponds to a parameter. If this parameter is not zero, it means that there is an edge between two nodes. Fig 4(c) plots the graph structure from *ℓ*_1_-regularization learning. Compare with the previous neighborhood structure in Fig 4(b), the learning structure of the graph based on the proposed method is sparse.

### 3.2 Image segmentation

The second group of experiments sought to test the effectiveness of the proposed algorithm for learning graph structure, and to implement image segmentation on BRATS and HNPPH clinical dataset.

#### 3.2.1 Feature learning

We firstly consider the problem of features extraction from image. Our image features set consists of four classes of features, namely intensity, texture, curvature, and fractal in here.

For each superpixel, we compute thirteen statistics features which are average, standard deviation, variance, mean of the absolute deviation, median absolute deviation, coefficient of variance, skewness, kurtosis, maximum, mode of the intensity values, central moments, range, interquartile range, and entropy.

RLCP inherits the advantages of LBP and GLRL features, and can be used to extract texture features in here. GLRL matrix is obtained from the indexed LBP image, which helps in extraction of textural information of brain tissues more thoroughly. LBP code is indexed and GLRL matrix in principle directions are formed to count occurrences of runs length for each gray level. Eleven features are calculated from literature [35]. Four directions (0°, 45°, 90°, 135°) GLRL matrix are calculated in each irregular patch of LBP image, this gives extracted feature vector of length 44(11 × 4).

The curvature features for each superpixel are calculated by Eq (7). The extracted curvature features are the average of the curvature values for all the pixels in the superpixel.

**Fig 5:**
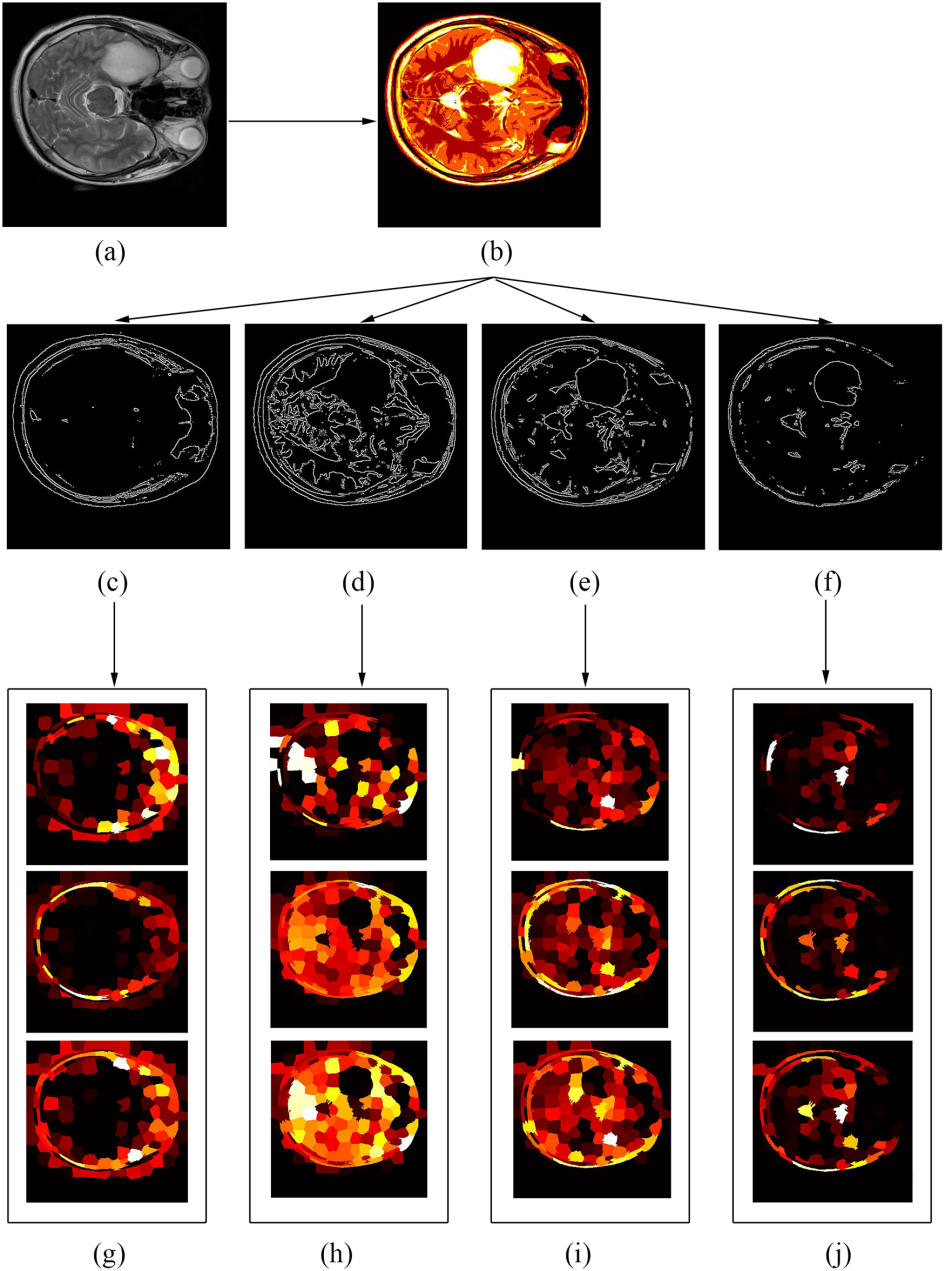
Fractal features. (a) Original image. (b) Otsu quantitatively image. (c)-(f) Four thresholded binary images obtained from (b). (g)-(j) Three features (area, mean intensity, and fractal dimension) from (c)-(f) respectively.

Experiment with 1–10 threshold levels for fractal feature extraction, after 4 threshold levels of threshold, the overlap measure is no obvious growth. In order to get better results and avoid time consumption, the optimum level of threshold 4 is chosen for fractal features. In summary, there are 12 fractal features obtained from 4 thresholded binary images (each binary image provides 3 fractal features) in Fig 5.

On the experiments, the parameters setting are carefully considered to obtain the optimal performance. For superpiexl, desired number of superpixels, compactness and number of iterations parameters need to be determined. For texture and fractal features, RLCP directions and binary images channel of fractal features should be selected for an accurate and fast calculate.

As shown in Table 1, since the resolution of images in the BRATS dataset is less than that in HNPPH dataset, we choose 40–200 numbers at 20 interval for superpiexl number on BRATS data set, and 40–260 numbers at 20 interval for superpiexl number on HNPPH data set. The best result is selected for superpiexl number, therefore, the number of superpiexl for each image can be different. The compactness parameter of the SLIC0 algorithm [40] controls the shape of superpixels. A higher compactness value makes superpixels more regularly shaped. The number of iterations used in the clustering phase of the algorithm is specified as 10. Four directions are calculated in RLCP for texture features, and 4 binary channel for fractal features.

**Table 1:** Parametric description.

Dataset	Superpiexl			Features		Optimization function
	Superpiexl number	Compactness	Number of iterations	RLCP directions	Binary channel	Max iterations number
BRATS15&13	40:20:200	40	10	0,45,90,135	4	50
HNPPH	40:20:260	40	10	0,45,90,135	4	50

Four class features are extracted from superpixel regions, however, not all of the features are what we want. We can choose the appropriate features and achieve image segmentation through the optimization of Eq (8). In optimization function, we use 50 as the maximum number of iterations.

#### 3.2.2 Image segmentation

**Fig 6:**
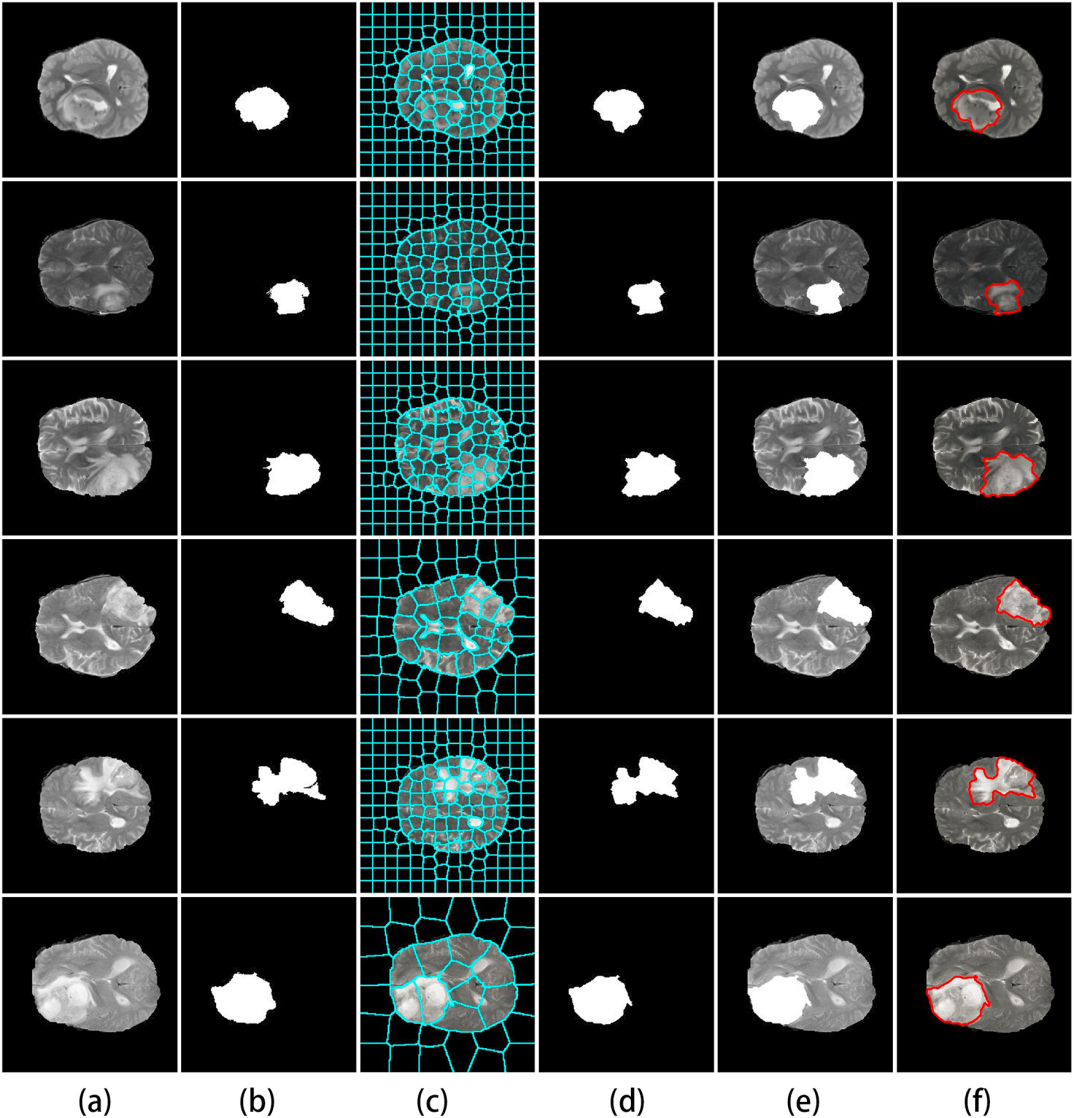
Results of HGG image segmentation in BRATS data set. (a) Original image. (b) Ground truth. (c) Superpixel segmentation. (d)-(f) Three demonstrations of segmentation results from the proposed method.

**Fig 7:**
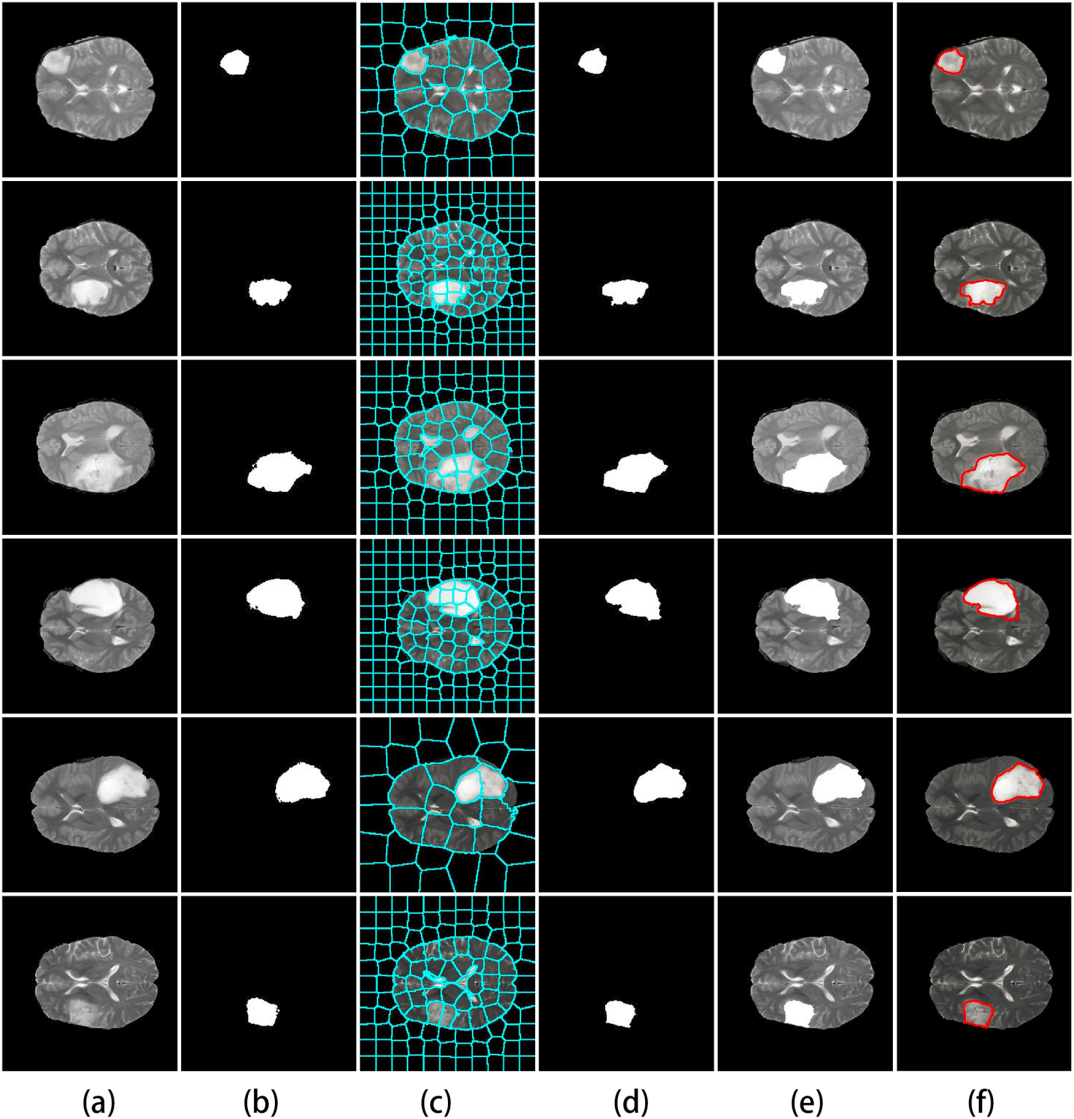
Results of LGG image segmentation in BRATS data set. (a) Original image. (b) Ground truth. (c) Superpixel segmentation. (d)-(f) Three demonstrations of segmentation results from the proposed method.

We conducted experiments with glioma segmentation on medical image data to for evaluating our segmentation approach. The proposed undirected graphical models are applied to glioma segmentation. We first test the proposed model on the BRATS data set for evaluating structure learning algorithm and implementing image segmentation. The HGG and LGG results are shown in Figs 6 and 7 respectively.

As shown in Figs 6 and 7, our image segmentation method is feasible. It also confirms the reliability of the results from the proposed method of learning structure.

**Fig 8:**
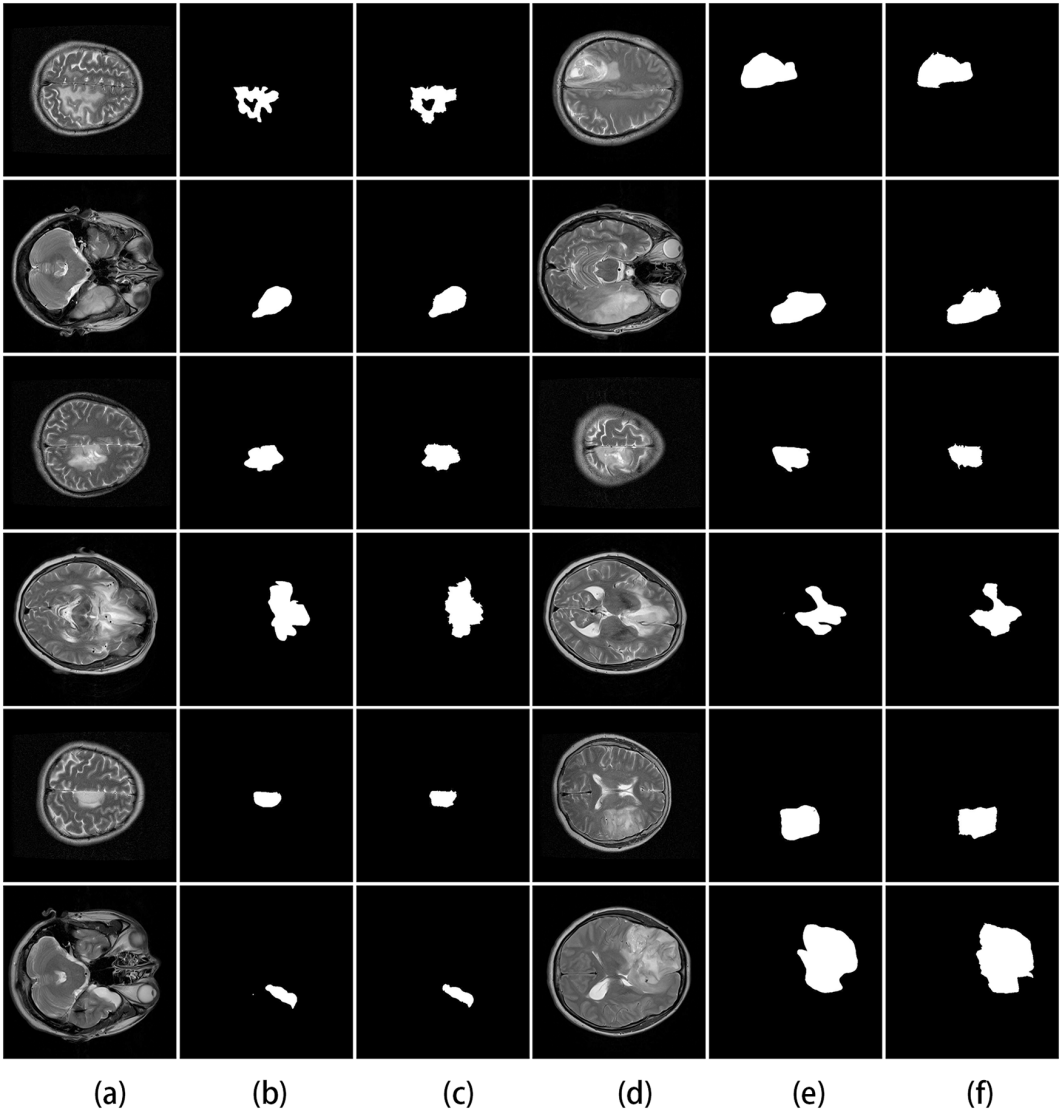
Results of HGG image segmentation in HNPPH data set. (a)&(d) Original image. (b)&(e) Ground truth. (c)&(f) Segmentation results from the proposed method.

**Fig 9:**
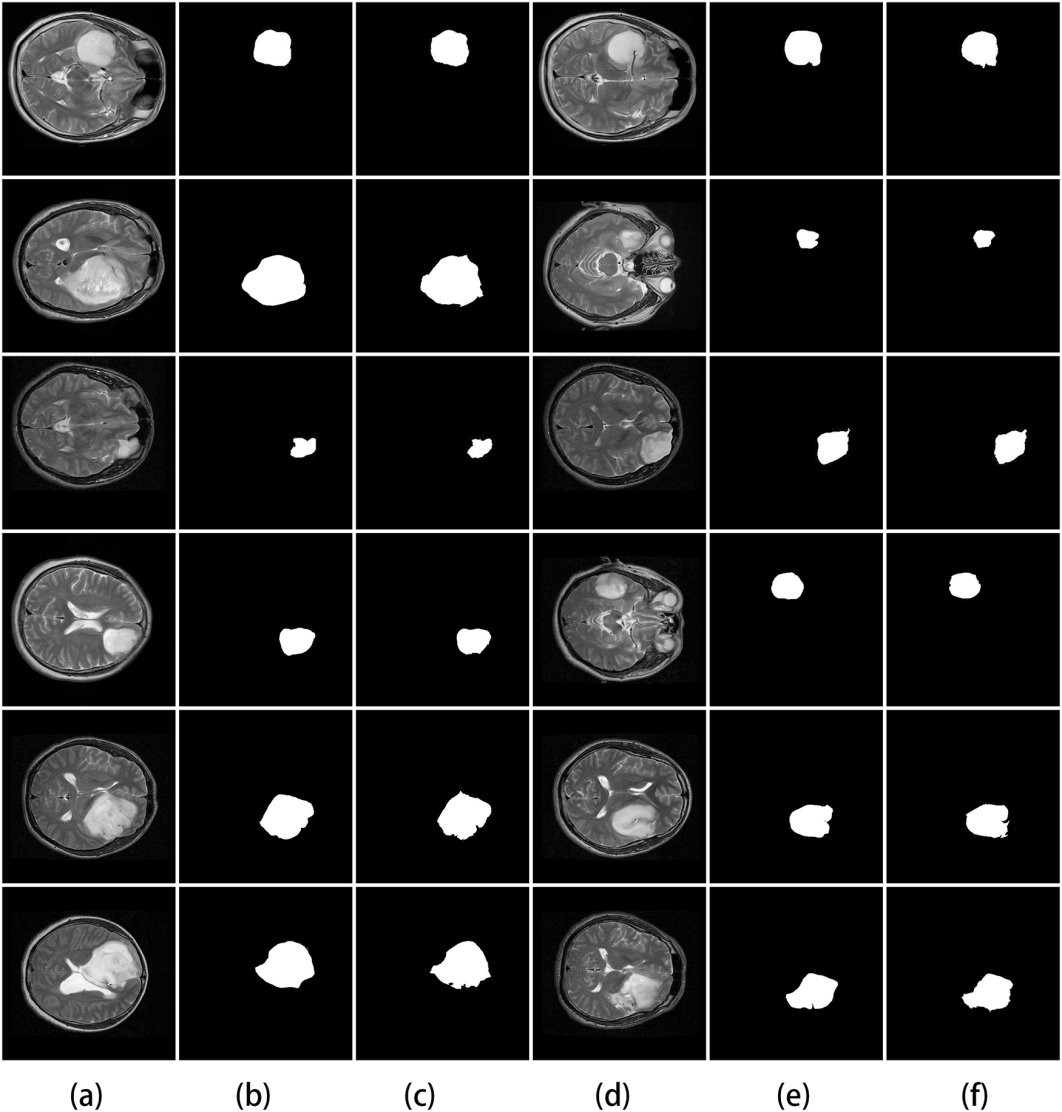
Results of LGG image segmentation in HNPPH data set. (a)&(d) Original image. (b)&(e) Ground truth. (c)&(f) Segmentation results from the proposed method.

In order to further test and verify the effectiveness of the proposed algorithm, we implement the image segmentation experiments on Henan Provincial People’s Hospital with 161 patients samples. Some HGG results shows in Figs 8 and 9 shows some LGG segmentation results.

The segmentation results of the proposed method are quite effective and accurate in most of the samples, but there are also some can not get acceptable result, for its reasons, generates superpiexls with not so good adherence to object boundaries in image, and the results of superpiexl are strongly correlated with the results of segmentation, this may adversely impact the performance. In future, we planned to improve the superpiexl segmentation approach to get better segmentation result.

We utilize five quantitative evaluation of the proposed model. Five evaluation criteria between the ground truth by data set provide and the method segmented ground truth using our method for the BRATS and HNPPH data set is presented in Table 2. Hausdorff Distance [41] is a mathematical construct to measure the “closeness” of two sets of points that are subsets of a metric space, the larger the result is, the more different the two sets are. If the Dice coefficient [41] value is 1 it shows perfect overlap if value is 0 there is no overlap between ground truth and segmented area. The correlation coefficient [42] (a value between -1 and +1) tells us how strongly two variables are related to each other, if it closed to 0 it means there is no relationship between the two sets. Specificity or true negative rate computes how much percentage of non tumor pixels correctly detected as non-tumor pixels. The range of metrics lies between 0 to 1 and maximal value is optimal. Sensitivity defines the percentage of tumor pixels correctly detected as tumor pixel. The range of metrics lies between 0 to 1 and maximal value is optimal.

**Table 2:** The quantitative evaluation of the proposed approach on data sets.

**LGG(Low-grade glioma)**					
**Dataset**	**Correlation**	**Dice**	**HausdorffDist**	**Sensitivity**	**Specificity**
Brats15&13	0.901	0.803	4.03	0.891	0.994
HNPPH	0.919	0.823	3.62	0.914	0.996
**HGG(High-grade glioma)**					
**Dataset**	**Correlation**	**Dice**	**HausdorffDist**	**Sensitivity**	**Specificity**
Brats15&13	0.912	0.813	3.65	0.893	0.995
HNPPH	0.923	0.823	3.46	0.915	0.996

We also test the proposed algorithm on the BRATS and HNPPH data set to compare the results with those obtained by three state-of-the-art image segmentation algorithms. The compared methods in this experiments include: Thiruvenkadam et al. [43], Zhao et al. [44], and Gu et al. [45].

**Fig 10:**
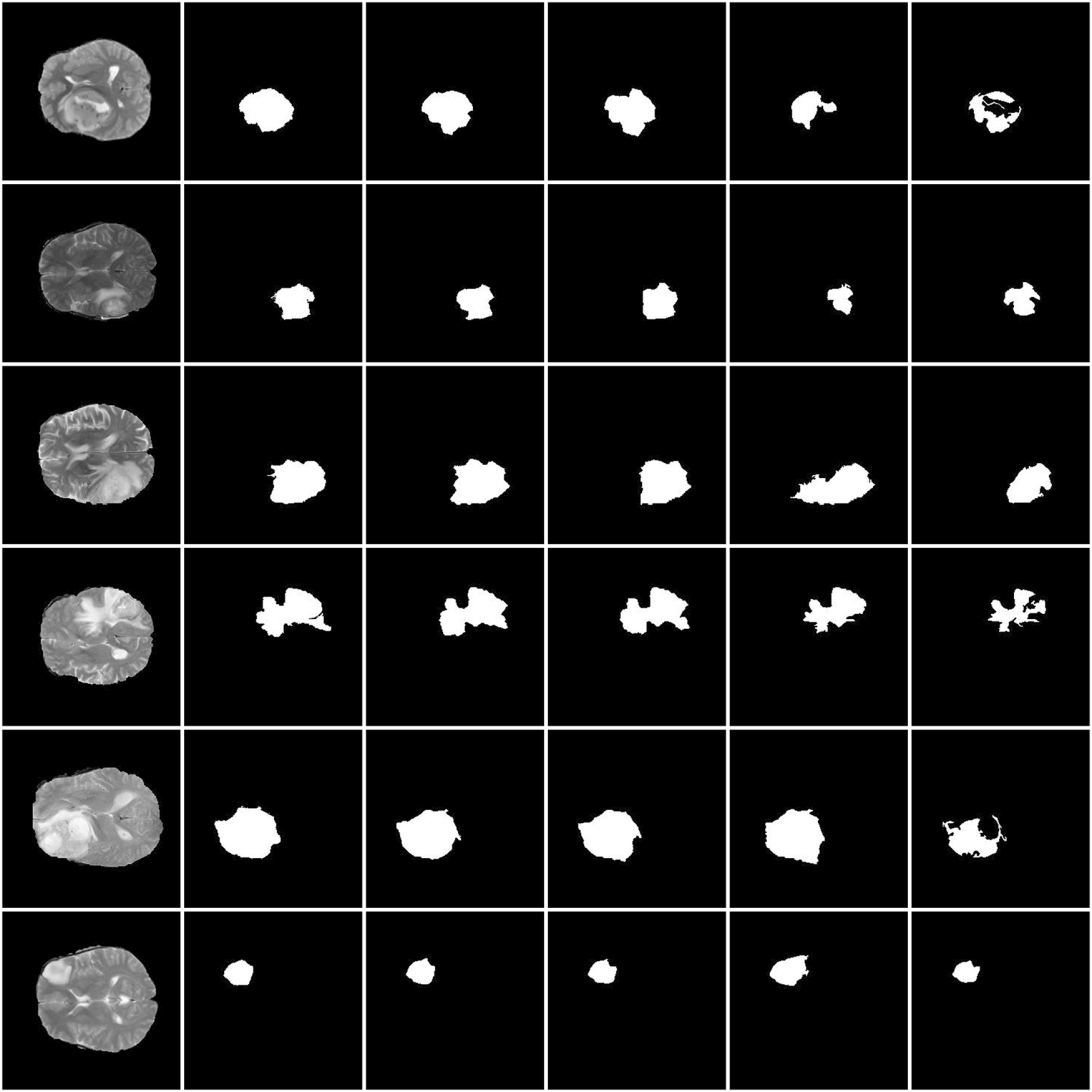
The comparison of our approach with three related works for segmentation in BRATS data set. The first column: the original images. The second column: ground truth. The third column: the proposed method. The fourth column: the method of Thiruvenkadam et al. The fifth column: the method of Zhao et al. The sixth column: the method of Gu et al.

**Fig 11:**
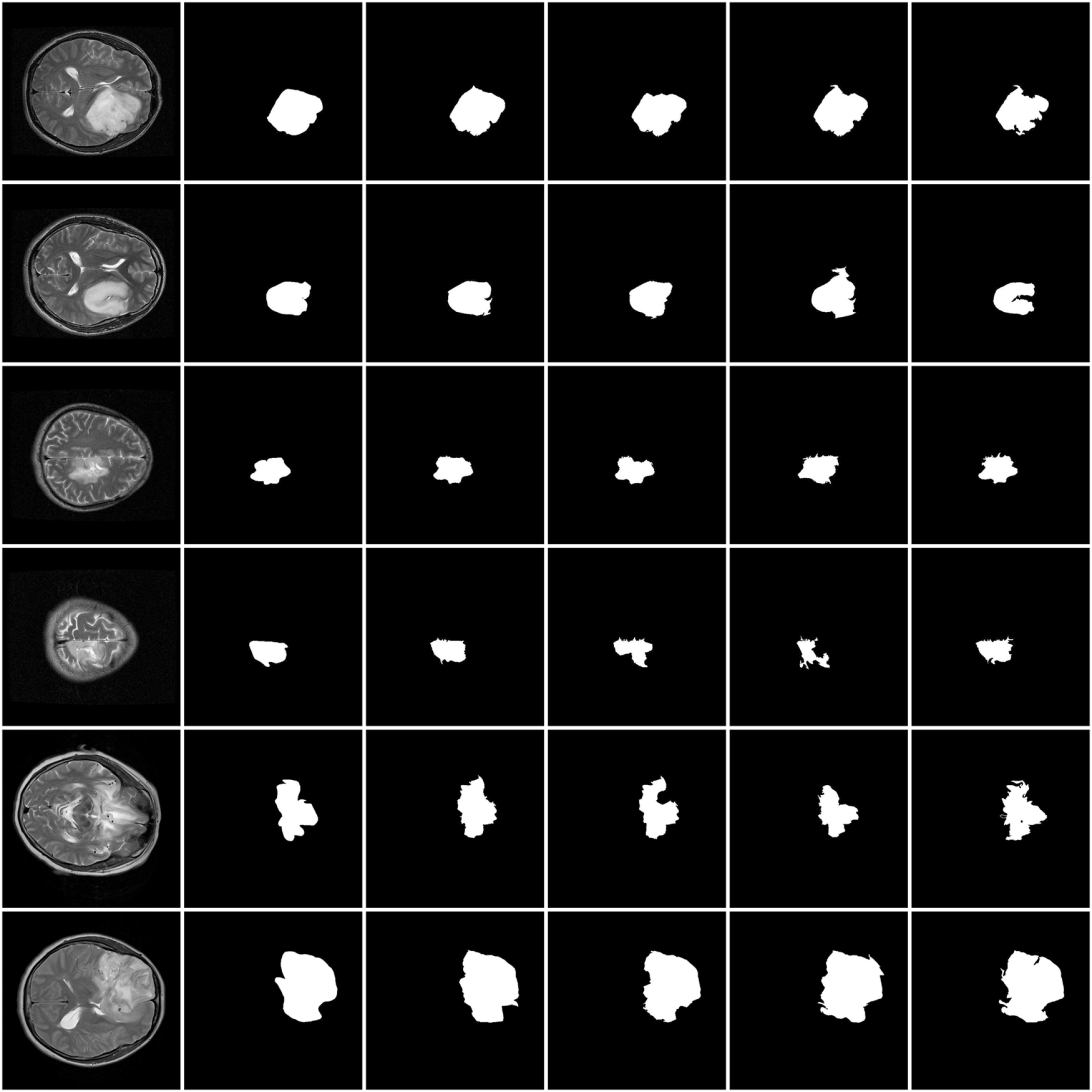
The comparison of our approach with three related works for segmentation in HNPPH data set. The first column: the original images. The second column: ground truth. The third column: the proposed method. The fourth column: the method of Thiruvenkadam et al. The fifth column: the method of Zhao et al. The sixth column: the method of Gu et al.

Fig 10 shows the proposed and existing methods output on the BRATS data set, and Fig 11 shows the proposed and existing methods output on the HNPPH data set, we observed that the proposed method (the third column) obtained satisfactory results of segmentation result than the other three methods. Some region which are wrongly classified by using other methods can be correctly classified by using the proposed method. The experimental results demonstrate the high detection and segmentation performance of the proposed method.

Empirical comparisons with several state-of-the-art algorithms demonstrate the efficiency of the proposed algorithm for image segmentation. Performance of the proposed method is compared with the existing methods for BRATS and HNPPH data set in the same computing environment, the results are summarized in Table 3.

**Table 3:** The quantitative comparison of our approach with three related works for segmentation on the two data sets.

References	Methods description	Dataset	Correlation	Dice	HausdorffDist
Thiruvenkadam et al.	DWT for pre- and post-processing, FCM for brain tissues segmentation	BRATS	0.865	0.785	4.35
		HNPPN	0.85	0.775	4.75
Zhao et al.	SLIC+ texture and merges, with spectral feature + PCA + SVM	BRATS	0.835	0.745	5.35
		HNPPN	0.78	0.685	5.9
Gu et al.	A single click ensemble segmentation (SCES) approach	BRATS	0.775	0.675	6.35
		HNPPN	0.84	0.765	5.25
**Proposed Methods**		**BRATS**	**0.905**	**0.805**	**3.8**
		**HNPPN**	**0.915**	**0.82**	**3.5**

The observed results show that our method can get higher Dice and Correlation values, as well as the smallest HausdorffDist. The performance results of the proposed methods is quite acceptable. In a word, our methods about structure learning and image segmentation can be shown to be effective.

## 4 Discussion

In this work we have introduced probabilistic framework for glioma segmentation. The novelty of our work is that we learn the structure of graphical models to incorporate them into a glioma segmentation framework based on conditional random fields. There are some considerations should factor into the performance of the glioma segmentation method in this work. One of them concerns with the quality of the superpixel algorithms. The reason for this is the accuracy of superpixel segmentation directly affects structure learning and image segmentation. It is difficult to obtain compact, regular superpixels, since compactness comes at the expense of boundary adherence. Nan et al. have reported good result by applying their method on superpixel segmentation [40]. This approach makes superpixels more regularly shaped. Another consideration is to integrate structural learning and image segmentation into a model. In other words, a objective function is constructed for the previous two tasks. We are currently exploring the possibility of incorporating structure learning, feature learning, and image segmentation into one model.

## 5 Conclusion

To summarize, we propose a novel glioma detection and segmentation framework based on structure learning of undirected graphical models. This method can achieve effective graph structure learning, and perform accurate glioma segmentation. The MRI images are first over-segmented into superpixel regions to reduce computational cost, and each superpixel is used to construct undirected graph models, after parameter optimization and structure learning, the graph structure is constructed. In glioma segmentation, base on this graph structure, we extracted the node and edge features for feature learning. Then, segmentation result of glioma is obtained through the optimization of the objective function. The experimental results demonstrate the proposed system improves the efficiency in glioma segmentation and makes it superior over the other existing systems.

## Supporting information

S1 File Samples from Henan Provincial People’s Hospital data set. This samples are provided by Henan Provincial People’s Hospital (HNPPH), there are one low-grade gliomas case (00 folder) and one high-grade gliomas case (11 folder), both case included T1 weighted imaging, T2 weighted imaging, fluid attenuated inversion recovery (FLAIR) imaging, enhanced T1 weighted imaging, and ground truth, and private information of all the samples was removed after collection.
(RAR)
